# SARS-CoV-2 Spike Protein and Long COVID—Part 1: Impact of Spike Protein in Pathophysiological Mechanisms of Long COVID Syndrome

**DOI:** 10.3390/v17050617

**Published:** 2025-04-25

**Authors:** Bruno Pereira de Melo, Jhéssica Adriane Mello da Silva, Mariana Alves Rodrigues, Julys da Fonseca Palmeira, Felipe Saldanha-Araujo, Gustavo Adolfo Argañaraz, Enrique Roberto Argañaraz

**Affiliations:** 1 Laboratory of Molecular Neurovirology, Department of Pharmacy, Faculty of Health Science, University of Brasília, Brasilia 70910-900, DF, Brazil; brunop.demelo@gmail.com (B.P.d.M.); jhessicaadrianems@gmail.com (J.A.M.d.S.); marianarodrigues0911@gmail.com (M.A.R.); julys.palmeira@hotmail.com (J.d.F.P.); gustaad2003@yahoo.com.br (G.A.A.); 2 Laboratory of Hematology and Stem Cells (LHCT), Faculty of Health Sciences, University of Brasília, Brasilia 70910-900, DF, Brazil; felipearaujo@unb.br

**Keywords:** long COVID syndrome, spike protein, pathophysiological mechanisms

## Abstract

SARS-CoV-2 infection has resulted in more than 700 million cases and nearly 7 million deaths worldwide. Although vaccination efforts have effectively reduced mortality and transmission rates, a significant proportion of recovered patients—up to 40%—develop long COVID syndrome (LC) or post-acute sequelae of COVID-19 infection (PASC). LC is characterized by the persistence or emergence of new symptoms following initial SARS-CoV-2 infection, affecting the cardiovascular, neurological, respiratory, gastrointestinal, reproductive, and immune systems. Despite the broad range of clinical symptoms that have been described, the risk factors and pathogenic mechanisms behind LC remain unclear. This review, the first of a two-part series, is distinguished by the discussion of the role of the SARS-CoV-2 spike protein in the primary mechanisms underlying the pathophysiology of LC.

## 1. Introduction

The enhanced transmissibility of the Severe Acute Respiratory Syndrome Coronavirus Virus 2 (SARS-CoV-2) is associated with severe clinical complications, including acute inflammation and disseminated intravascular coagulation [1]. Moreover, the emergence of more infectious variants of concern (VOCs) has been one of the significant factors contributing to the high rates of morbidity and mortality observed during the COVID-19 pandemic [2]. Vaccination and treatment advances have significantly reduced the severity and mortality of COVID-19 and the transmission of the virus [3]. Nevertheless, over the past three years, up to 40% of recovering patients have experienced long COVID syndrome (LC) or post-acute sequelae of COVID-19 (PASC) [4]. In the current scenario of global SARS-CoV-2 infection, characterized by the continuous emergence of VOCs and the rising incidence of LC, it is a priority to understand the underlying molecular mechanisms of LC. This review was developed as a scoping or conceptual review, using specialized literature databases for critical support. This is the first part of a two-part series that will address the relevance of the SARS-CoV-2 spike (S) protein to the main pathophysiological mechanisms of LC. Moreover, it is essential to emphasize that this review aims to provide a comprehensive examination of the role of the S protein in the pathophysiological mechanisms of LC, grounded in a critical and systematic approach to literature selection.

## 2. Long COVID/PASC: A Chronic Disease?

LC has become a growing public health problem, affecting at least 65 million people worldwide [5]. According to the WHO, LC refers to the continuation or development of new symptoms lasting at least three months following the initial viral infection. It may persist for two or three years, with symptom exacerbations over time, though determining the permanence of symptoms is difficult due to the lack of standardized research methods [6,7,8]. In general, types of LC are defined according to the severity and duration of symptoms [9,10]. Recovery from the acute phase is typically slow, but most symptoms decrease over time and stabilize around 6–8 months after onset. In a French cohort study, it was found that 10% of all patients who had been infected with COVID-19 experienced LC. Additionally, among those who were symptomatic, 85% reported ongoing symptoms at least one year later, with prevalence varying among individuals [5]. This percentage was 50–70% for non-hospitalized patients and 10–12% for vaccinated patients [11,12]. Nevertheless, in a recent study with 135,161 patients, the risk of death in hospitalized patients remained for three years, and the risk of LC increased [13].

LC is characterized by several long-lasting symptoms, the most common being dyspnea, fatigue, headache, cognitive dysfunction, myalgia, joint pain, loss of smell and taste dysfunction, cough, insomnia, and rhinorrhea, among others [14]. Less common symptoms include chills, flushing, hair loss, ear pain, visual impairment, heart and gastrointestinal issues [15,16]. Neurological manifestations may include encephalopathy, stroke, seizures, and Guillain–Barre Syndrome. In a study conducted with 135 LC patients with different clinical outcomes, 15% of patients developed one or more neurological symptoms [17]. Among these symptoms, it is worth highlighting that 13% had polyneuro/myopathy, 1% had Guillain-Barré syndrome, 2% had mild encephalopathy, 1% had parkinsonism, and 1% had ischemic stroke [17]. A meta-analysis of 257,348 patients with post-acute COVID-19 showed that symptoms and prevalence depended on the period analyzed [18]. Between 3 and 6 months, fatigue (32%), dyspnea (25%), sleep disturbance (24%), and difficulty concentrating (22%) were the most common symptoms; between 6 and 9 months, exertion intolerance (45%), fatigue (36%), sleep disorders (29%), and dyspnea (25%); between 9 and 12 months, fatigue (37%) and dyspnea (21%); and beyond 12 months of follow-up, fatigue (41%), dyspnea (31%), sleep disorders (30%), and myalgia (22%) were the most common. Furthermore, in a study of 10,530 long COVID patients followed for at least three months, the most common neurological symptoms included fatigue (37%), dysgeusia (10%), brain fog (32%), attention disorder (22%), myalgia (28%), memory issues (28%), anosmia (12%), and headaches (15%), and these persisted for up to six months or one year [19]. In a retrospective study of 236,379 patients recovering from COVID-19, neurological and psychiatric morbidities were detected in 33.62% of patients six months after COVID-19 infection [20]. Moreover, the risks were higher in patients with severe COVID-19, with, for example, the estimated incidence for patients admitted to the ICU being 46.42%. Nevertheless, it is important to emphasize that age is a crucial risk factor for the development and outcomes associated with LC. As outlined, the development of LC is multifaceted and involves physiological, psychological, and immunological factors, which are closely linked to aging. Indeed, age-related changes in lung function, cardiovascular health, and neurological resilience may influence the prolonged and varied symptoms presented by patients with LC [21,22,23].

The types and severity of COVID-19 symptoms can also depend on the SARS-CoV-2 variant [9,10]. The prototypical variant (Wuhan) appears to induce more symptoms than the Alpha or Delta variants [24]. Furthermore, a prospective study of hospitalized LC patients demonstrated that the Omicron variant initially led to greater symptom severity, in addition to the Delta lineages AY.126 and AY.43, and the Omicron sublineages BA.1.17, BA.2.56, and BA.5.1 consistently correlated with more severe disease symptoms [25]. These data demonstrate how specific lineages can influence the progression and severity of LC sequelae [26]. In summary, the variability in incidence rates and symptom diversity indicate, a priori, that LC is a progressive, multiphasic, and multisystemic disease with a significant impact on quality of life [4].

## 3. The Pathophysiology of Long COVID and the Spike Protein

COVID-19 is a multisystem disease characterized by intense inflammation and disseminated intravascular coagulation [13]. It remains unclear whether specific mechanisms are responsible for certain symptoms or whether the long COVID symptoms result from a combination of mechanisms. The leading hypotheses include the following: (i) chronic inflammation driven by the persistence of viral components in reservoirs [27]; (ii) immune-dysregulation-mediated inflammation and autoimmunity [28,29]; (iii) the reactivation of latent viral infection [30]; (iv) complement dysregulation and thromboinflammation [31]; (v) the dysbiosis of the microbiome [32]; (vi) unresolved tissue damage [5]. Furthermore, recent evidence has also led to new hypotheses/mechanisms to explain the genesis of LC. In this regard, reduced intracerebral acetylcholine, the reduction in serotonin, and the change in iron metabolism stand out [33,34]. However, most hypotheses have underlying mechanisms of persistent inflammation and endothelial dysfunction and damage, followed by thrombi formation and coagulation activation. Indeed, endothelial damage and activation may lead to platelet aggregation, monocyte clustering, and tissue factor (TF) expression in the vascular wall, culminating in thrombus formation and the activation of the extrinsic coagulation pathway [35,36].

## 4. Viral Reservoirs

**Figure 1:**
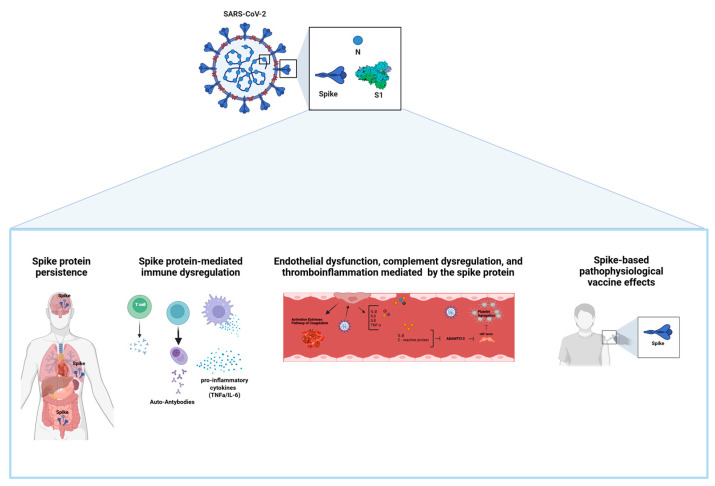
The pathophysiological effects mediated by the SARS-CoV-2 S protein in long COVID. The pathophysiological outcomes associated with the SARS-CoV-2 spike protein include: (i) viral component persistence; (ii) immune system dysregulation leading to increased levels of pro-inflammatory cytokines and autoantibodies; (iii) endothelial dysfunction, complement system imbalance, and thromboinflammation; (iv) pathophysiological effects related to spike protein-based vaccines.

Viral persistence-mediated inflammation is one of the main mechanisms cited in the pathophysiology of LC [27]. Viral components (RNA and proteins) can be detected by multiple pattern recognition receptors (PRRs), leading to the production of inflammatory cytokines such as TNF-α, IL-1β, IL-6, and IL-18, type I and III interferons, and chemokines [37,38]. In addition, the repeated recognition of persistent viral components could lead to the activation, exhaustion, and altered differentiation of viral-specific T and B cells [39]. SARS-CoV-2 components (S, spike subunit S1, and nucleocapsid [N] proteins) can remain in circulation and across various systems and tissues for a considerable time [40,41]. Indeed, SARS-CoV-2 components have been identified in the respiratory, cardiac, renal, reproductive, and central nervous systems (CNS), as well as in lymph nodes, muscles, the liver, and the gastrointestinal tract (GI) [27,42,43]. In a recent study analyzing almost 94,000 viral sequences to rule out reinfection cases, and from the follow-up of 381 individuals, it was observed that up to 0.5% of SARS-CoV-2 infections may become persistent for at least 60 days, usually with viral rebounds [44]. Individuals with viral persistence had a greater than 50% likelihood of developing long COVID, with 30% experiencing viral rebounds. Most cases of viral persistence were resolved in less than three months, although not all cases resulted in LC (only 9% did). Furthermore, in an autopsy study of 44 patients, the persistent presence of SARS-CoV-2 RNA was detected at various anatomical sites, including the brain, cervical spinal cord, and olfactory nerve, persisting between 31 and 230 days after the onset of symptoms [45,46]. However, little evidence of inflammation or viral pathogenesis was observed beyond the respiratory tract, and persistence was found to be time-dependent and symptom-dependent [27]. Other groups detected the persistence of viral components in different tissues, for several periods after acute disease, in the colorectal tissue (158–676 days) [47], olfactory mucosa (110–196 days) [48], and skin/appendix/breast (163–426 days) [49]. In another study, conducted with 317 tissue samples collected from 225 patients at three different time points after mild COVID-19 infection, the presence of SARS-CoV-2 viral genomic and subgenomic RNA was detected in multiple tissues (liver, kidney, stomach, intestine, brain, blood vessels, lung, breast, skin, and thyroid) up to 4 months after infection [50]. Importantly, viral RNA was detected in the plasma and peripheral mononuclear blood cells (PBMCs) of immunocompromised patients, but not in immunocompetent ones. Notably, the prevalence of viral RNA was significantly associated with the development of LC symptoms, with patients having higher viral copy numbers showing a higher probability of developing long COVID symptoms [50]. Gut enterocytes are particularly susceptible to SARS-CoV-2 infection and replication [51]. In a study of intestinal biopsies collected 4 to 6 months after an acute SARS-CoV-2 infection, the N protein and viral RNA were detected in the intestinal epithelium of one-third of the individuals, even in the absence of evidence of viral replication in a nasopharyngeal swab [52]. Furthermore, Zollner et al. reported the presence of SARS-CoV-2 RNA and N protein in the gut mucosa for approximately seven months after mild acute COVID-19 (69% and 52%, respectively) [53]. However, the persistence of viral components was associated with PASC symptoms but not with the severity of acute COVID-19 or inflammation [52]. These findings suggest that shared symptoms in both acute COVID-19 and LC, particularly inflammation and immunological disorders, could be driven by two underlying mechanisms: ongoing viral replication and the presence of viral components even in the absence of active replication [54] (Figure 1).

### Persistence of the S Protein

Most studies investigating the viral persistence of components after the acute phase of COVID-19 have shown that patients with LC exhibit sustained and elevated levels of circulating glycoprotein S for up to one year or more after the initial infection [41]. The presence of viral components in circulation is mainly related to free S1 protein or its incorporation within extracellular vesicles (EVs), along with viral RNA. This phenomenon is more prominent in LC patients than in convalescent control patients [41]. The S protein may be stored in cellular reservoirs, such as adipocytes, and released into circulation in EVs, subsequently taken up by tissue cells, such as cardiomyocytes and pericytes, leading to complications such as cardiomyopathy [55]. Although the origin of tissue/reservoir EVs remains unclear, they may help to avoid the immune response and facilitate the transport of SARS-CoV-2 S proteins from reservoir tissue sites into the circulation or from cell to cell, such as from astrocytes to neurons [47]. The detection of S protein in the plasma of PASC patients for more than one year could be due to ongoing viral replication or, alternatively, reflect waves of immune control success and failure over time [41]. Recent studies have addressed several of the limitations of previous studies, including low population representation, limited follow-up, a lack of clear data on vaccination or reinfections, and comparison with a group of truly negative patients. However, a recent study with 171 plasma samples collected before vaccination and the emergence of the Delta and Omicron variants demonstrated that the most commonly detected antigens were S, S1, and N proteins at different time points during the 14 months after SARS-CoV-2 infection [40]. Interestingly, patients who required hospitalization were almost twice as likely to have antigens detected in the post-COVID-19 phase. Another study using an optimized ultrasensitive single-molecule array (Simoa) method was able to detect S, S1, or N proteins in approximately 65% of plasma samples collected from LC patients up to 12 months after SARS-CoV-2 infection and before receiving any SARS-CoV-2 vaccine [41]. This body of evidence strongly suggests that the severity of symptoms in the post-acute phase is modulated, at least in part, by the persistence of SARS-CoV-2 reservoirs and, mainly, by the S, S1, and N proteins found in several tissues and circulation [27]. However, it is important to highlight that viral component persistence does not occur in all individuals with long COVID-19, and there is a percentage of patients who do not present PASC symptoms, even when the remaining viral components are present. Therefore, the real significance and impact of the persistent presence of the S protein, specifically the S1 subunit, in both PASC and convalescent patients require further research, mainly in relation to the host’s genetic background. In this context, a recent study utilizing single-cell multi-omics profiling identified HLA-DQA2 as a protective allele against long COVID [56].

## 5. Epithelial and Endothelial Dysfunction

Endothelitis and thrombosis–inflammation have been observed in the acute and post-recovery phases of COVID-19 [57]. Severe cases of COVID-19 are often associated with comorbidities such as advanced age, obesity, diabetes, hypertension, and cardiovascular diseases, all of which are characterized by chronic endothelial dysfunction [58]. SARS-CoV-2 can cause endothelial cell (EC) damage and disrupt the pulmonary endothelial cell barrier, leading to edema, hyperinflammation (cytokine storm syndrome), hypercoagulability, and severe thrombosis, which are critical features of COVID-19 [59,60]. EC injury or dysfunction, endothelitis, and vasculitis-mediated thrombo-inflammation can occur through multiple pathways, including viral infection, spike–receptor interaction, or cytokine exposure, and may persist after the acute phase of COVID-19 [61,62]. The primary mechanisms of EC dysfunction include the following: (i) SARS-CoV-2 infection-mediated apoptosis; (ii) the renin–angiotensin system (RAS)/kallikrein–kinin system (KKS) imbalance; (iii) complement activation; (iv) activation of inflammatory, mitochondrial oxidative stress, and growth factor signaling pathways. These mechanisms, individually or in combination, can lead to endothelial damage and are linked to an increased risk of long COVID [35,63]. Additionally, the failure of ECs to release sufficient amounts of nitric oxide (NO) can result in vessel constriction and a reduced ability to neutralize reactive oxygen species (ROS) and viral replication [64]. ROS generated from mitochondria also contribute to oxidative stress and appear to play a role in the pathogenesis of COVID-19 [65]. Indeed, acute COVID-19 inflammation can lead to excessive ROS production and cell apoptosis, mediated by cytochrome-c; the activation of the calcium and NF-kB signaling pathways; an increase in vascular permeability; and the promotion of leukocyte adhesion [66]. Moreover, oxidative stress caused by NOX2 activation has been associated with thrombotic events in COVID-19 patients [67].

The IL-6/IL-6R pathway activation, triggered by ADAM17, can also induce endothelial activation/dysfunction [68] and reduce NO levels, increasing oxidative stress and adhesion molecule expression, leading to leukocyte recruitment and vascular permeability [69]. Regardless of the mechanism involved, endothelial dysfunction results in pathophysiological changes that culminate in inflammatory immune cell infiltration, vascular leakage, and edema, known as “vascular long COVID”. Thus, vascular long COVID results in the secretion of inflammatory cytokines (TNF-α, IL-1β, IL-6) and chemokines, adhesion molecules (ICAM-1, VCAM-1, MCP-1, VAP-1), AngII-AT1R activation [70], IL-6/ROS production [71], KKS-B1/2R activation, and increased levels of vascular endothelial growth factor receptor (VEGF) and VEGFA/VEGFR2 activation [72].

### S-Protein-Mediated Epithelial and Endothelial Dysfunction

Epithelial and endothelial dysfunction has been linked to the interaction between the S protein and cellular receptors, independent of viral infection [35]. The S protein can induce barrier dysfunction and vascular leak/vasculitis via a mechanism independent of viral replication and the angiotensin-converting enzyme 2 (ACE2) receptor [73]. Conversely, the interaction between the S protein and integrins triggers transcriptional responses related to extracellular matrix reorganization and the TGF-β signaling axis [73]. Although supraphysiological concentrations of recombinant S protein (10 µg/mL) were used, in another work conducted with human pulmonary artery smooth muscle and endothelial cells, just 10 ng/mL of recombinant SARS-CoV-2/S1 subunit was sufficient to trigger a rapid and transient MEK phosphorylation with a peak at 10 min [74].

Interestingly, the ACE2-S binding domain (S-RBD) was not sufficient to induce MEK activation, since pre-incubation with the anti-ACE2 antibody did not completely inhibit S1-mediated MEK activation, suggesting the participation of other S1 subunit regions. The activation of cell growth and the MEK/ERK pathway mediated by the S1 subunit protein was correlated with the thickening of pulmonary vascular walls, potentially promoting hypertrophy in vascular smooth muscle cells, endothelial cells, and facilitating viral replication [75]. These findings are supported by the thickened alveolar septa and increased infiltration of mononuclear cells in Syrian hamsters inoculated with S-pseudovirus [76]. Furthermore, the S protein alone can induce endothelial damage by impairing eNOS activity and mitochondrial function, which results in increased redox stress, AMPK deactivation, MDM2 upregulation, and ACE2 downregulation. These findings were confirmed through in vivo and in vitro studies, demonstrating that the S protein activates the endothelial cell inflammatory phenotype through integrin α5β1 signaling [77]. Viral infection-related calcium increase is well established and essential for the viral lifecycle (entry, replication, maturation, and release) [30]. Calcium channel induction and calcium homeostasis dysregulation are important factors for viral infection and transduction signals involved in apoptosis and other cellular disorders. The role of increased intracellular calcium in SARS-CoV-2 infection and the pathogenesis of COVID-19 was further confirmed by later studies, which demonstrated that the S-RBD-ACE2 interaction activates calcium channels, leading to increased intracellular calcium levels. The increased calcium levels, in turn, lead to TMEM16F or ANO6 scramblase activation, and subsequent phosphatidylserine (PtdSer) exposure on the outer leaflet of the cell membrane, confirming our previous hypothesis [61,78].

Interestingly, the prototypical Wuhan strain induces more apoptosis than the Omicron variants (BA.5.2 and XBB), Delta, or Lambda, highlighting the possible role of the S-RBD domain in vascular pulmonary dysfunction [78]. Additionally, the S protein, RBD domain, or integrin-binding tripeptide RGD can induce NF-κB activation, leading to leukocyte adhesion molecules (VCAM1 and ICAM1) and EC monolayer hyperpermeability, along with the expression of coagulation factors (TF and FVIII), pro-inflammatory cytokines (TNF-α, IL-1β, and IL-6), and ACE2 [77]. Furthermore, ACE2-mediated SARS-CoV-2 S-pseudovirus transduction induces autophagy, inflammatory responses (IL-6, IL-8, and TNF-α), and apoptosis via ROS upregulation and PI3K/AKT/mTOR pathway inhibition in bronchial epithelial and microvascular endothelial cells [79]. Interestingly, ECs exposed to culture medium from A549 cells expressing the SARS-CoV-2-S protein exhibited markers of senescence, adhesion molecules, and ROS activation [80]. A later study showed that the S protein triggers the expression of pro-inflammatory cytokines and chemokines in a dose-dependent manner, but not IFN expression, in macrophages and lung epithelial cells [81]. Conversely, transfected cells expressing S protein did not induce an inflammatory response but elicited an inflammatory response in a paracrine manner and depended on the TLR2/MyD88/NF-κB pathway [81]. Moreover, several pieces of evidence support the role of S protein and S1 subunits in pulmonary vasculature activation in both in vitro and in vivo models [82,83]. Pericyte cells (PCs) support and provide maintenance and repair to cardiac microvasculature and also express high levels of ACE2 and CD147 SARS-CoV-2 receptors in the heart [84]. Reduction in heart and lung PCs in COVID-19 patients suggests that viral infection may destabilize microvasculature by direct PC infection [85]. Interestingly, the Wuhan strain and the Delta and Omicron variants did not cause significant infection in primary cardiac PCs, and there was no correlation with ACE2 level expression. Nevertheless, PCs treated with recombinant protein S (1 µg/mL) exhibited notable functional and signaling alterations, such as enhanced migration, a reduced ability to facilitate EC network formation (PC-EC interaction), an increased secretion of pro-inflammatory cytokines, and an elevated production of pro-apoptotic factors, ultimately resulting in EC death. These effects were mediated by the CD147 receptor instead of ACE2 and involved activation (ERK1/2) [86]. This study suggests that S-protein-mediated PC dysfunction may contribute to microvascular injury.

This body of evidence strongly suggests that the S protein and S1 subunit may play a critical role in the development of vascular long COVID across various biological systems [87] (Figure 1).

## 6. Immune-Dysregulation-Mediated Inflammation and Autoimmunity

Different types of immune-dysregulation-mediated inflammatory responses have been extensively documented in the pathophysiology of acute COVID-19 and LC [29,88]. Indeed, distinct immunological signatures in LC patients with pulmonary or neurological symptoms are characterized by the higher production of inflammatory cytokines, mainly IL-6, TNF-α, and IL-1β, along with a reduced CD8+ T cell activation diversity and exhaustion markers expression (PD-1 and CTLA-4, respectively) in SARS-CoV-2 spike-specific CD4+T cells [88,89,90,91]. A recent study found that IFN-γ-producing T cells are the primary contributors to a profibrotic response from monocyte-derived macrophages, alongside abnormal interactions between lung macrophages and resident respiratory T cells observed in bronchoalveolar lavage fluid from patients with long COVID and mice. [92,93]. More recently, the IFN-γ +874T/A SNP was associated with susceptibility to symptomatic COVID-19 [94]. On the other hand, numerous studies have linked the persistence of low levels of virus replication or non-replicative forms (RNA and proteins) in sanctuaries with sustained immune activation, T cell depletion, and specific inflammatory mediator patterns. Dysregulation of LB function during COVID-19 has been linked to the production of autoantibodies against interferon, neutrophils, connective tissue, and other cellular components [28].

Additionally, the dysfunction of T cells and antigen-presenting cells leads to autoreactive T cell activation and the infiltration of CD8+ T cells in several organs [95]. Immune dysregulation has also been found in LC patients, marked by exacerbated and exhausted CD4+ and CD8+ T cell responses [88]. Insufficient or inappropriate local LT cell activation during the acute phase may explain the inability to eliminate residual gastrointestinal infection and the reactivation of other viruses, such as Epstein–Barr virus (EBV) [96,97,98]. Nevertheless, an excessive response in the acute phase can trigger autoimmune reactions [99]. Moreover, in a recent study involving 148 individuals, including 87 with acute COVID-19 and 61 in the post-COVID-19 period, increased cGAS and STING gene expression, along with elevated IFNα levels, were associated with long-term COVID-19 [99]. These findings indicate that this pathway remains activated in the tissues of patients with long-term COVID-19, and it may play a critical role in the autoinflammatory disease observed in these individuals. Notably, recent research involving a group of LC patients, who were recruited before vaccination and displayed similar symptoms, identified two contrasting types of antiviral immune responses: one characterized by adequate antibody and CD4+ T cell responses and the other lacking these responses. These findings suggest that multiple, possibly interconnected mechanisms are involved in the pathophysiology of long COVID. [100]. Furthermore, SARS-CoV-2 infection can induce cross-reactive antibodies with host proteins through molecular mimicry, with mammalian heart, gut, lung, kidney, and brain self-antigens potentially sustained by the presence of a SARS-CoV-2 reservoir [101]. The relationship between the reactivation of EBV infection and molecular mimicry between the EBNA1 protein from EBV and the CNS protein GlialCAM suggests the involvement of a cross-reactivity mechanism [102]. Curiously, a decrease rather than an increase in levels of autoantibodies against chemokines has been recently reported in long COVID patients compared to recovered COVID-19 patients [103]. To date, no consistent autoantibody pattern in LC has been detected [104]. Taken together, these findings strongly indicate that the presence of viral components could lead to the prolonged elevation of interferons (IFNs) and cytokines, and certain autoantibodies may create conditions that allow for such viral persistence. However, a definitive causal link between these factors has not yet been established [27].

### S-Protein-Mediated Immune Dysregulation

A cohort study was conducted, involving 230 patients classified into different groups according to disease severity and the presence of antibodies against SARS-CoV-2 S proteins, including RBD and S1, as well as spike proteins from other coronaviruses, such as SARS-CoV and 229E. SARS-CoV-2 S protein was associated with inflammatory dysregulation in cytokine microenvironments and apoptotic factors, among other activities. However, without a defined pattern or a clear correlation between the anti-spike antibody response of the different coronaviruses and the severity of the disease. The humoral responses to anti-S Abs have also shown no differences between patients with and without LC, nor in the initial Ct-PCR value for the virus [105,106]. Thus, it becomes challenging to determine whether recent seasonal coronavirus infections may influence or attenuate the inflammatory response and/or clinical course of SARS-CoV-2 infection [107].

## 7. Complement Dysregulation and Thromboinflammation

The complement system plays a crucial role in humoral immunity, working alongside antibodies to eliminate invading microorganisms. However, if left unchecked, it can result in cellular and vascular damage. During the acute phase, antigen–antibody complexes and lectins (which identify specific carbohydrate structures on the SARS-CoV-2 spike protein) primarily activate the complement classical and lectin pathways, respectively [108]. Cervia-Hasler et al. detected high levels of C5b-C6 in the serum of long COVID patients, indicating excessive complement activation. Moreover, they also noted the upregulation of coagulation factors such as pro-coagulation Factor XI (F11) and von Willebrand factor (vWF), alongside a decrease in disintegrin and metalloproteinase with thrombospondin motif 13 (ADAMTS13), which is responsible for cleaving vWF multimers [109]. The dysregulation of the complement system, followed by an increase in vWF multimers, may affect normal endothelial function, facilitating platelet aggregation and the formation of clots. Supporting the role of complement in the pathogenesis of long COVID, Proterious et al. identified circulating microclots resistant to fibrinolysis enriched in vWF, C7, acute-phase serum amyloid proteins, and fibrin in samples from patients with long COVID [31]. Additionally, autopsies of brain tissue from patients who died due to SARS-CoV-2 infection reveal activation of and damage to the microvasculature, mediated by immune complexes (ICs) and complement activation, leading to the deposition of platelet aggregates and microthrombi in the endothelium [110].

### S-Protein-Mediated Complement Activation

The infusion of the S protein in the brain of mice was shown to be sufficient to affect cognitive function in mice, through complement-mediated synapse destruction, recapitulating post-COVID-19 syndrome [111]. Damage to the blood vessels that support the Blood–Brain Barrier (BBB), caused by the formation of immune complexes and complement activation mediated by the S protein, has been identified as a key factor in the brain damage and chronic neurological symptoms associated with LC [112]. These findings provide compelling evidence that the persistent circulation of the S protein may be a major risk factor directly related to the pathogenesis of Neuro-PASC (Figure 1).

## 8. Additional Long COVID-19 Mechanisms

### 8.1. Dysbiosis of Microbiome

Similarly to other viral infections, such as HIV-1 infection, the dysbiosis of the microbiota plays a significant role in the onset and progression of COVID-19 and long COVID [113,114,115]. In fact, early reports related to the Wuhan variant indicated that gastrointestinal symptoms, such as nausea and diarrhea, were present in patients with acute COVID-19 [116,117]. Notably, the alteration in microbial signatures in patients with COVID-19 was distinct from what was observed in those infected with the H1N1 strain of the influenza virus [118]. Patients with COVID-19 exhibited a marked decrease in bacterial diversity, including symbiotic species, along with an increase in opportunistic pathogens, such as Collinsella, Streptococcus, Ruminococcus, and Bacteroides, among others. Furthermore, the presence of *Clostridium innocuum* and *Actinomyces naeslundii* was correlated with persistent respiratory symptoms, while others showed a reverse correlation with PACS [119,120,121]. Alteration in the gut microbiome during acute COVID-19 has important consequences, leading to an increase in its permeability for harmful substances, inflammation, and the dysfunction of the microbiota–gut–brain axis [122]. In this context, a gut–lung-axis-mediated mucosal immune response can be induced by microbial dysbiosis [123,124]. The imbalance of these microorganisms, or dysbiosis, can persist for up to a year after infection [53,119,121]. A proof-of-concept of the involvement of microbial dysbiosis in the genesis of PASC symptoms was obtained through fecal transplantation of material from post-COVID individuals into germ-free mice, which resulted in lung inflammation and worse outcomes during multidrug-resistant Klebsiella pneumoniae lung infection, in addition to low cognitive performance [32]. However, there are no data to support the direct involvement of the spike protein in the imbalance of the gut microbiota. Overall, the data from the literature suggest that intestinal dysbiosis may directly contribute to COVID-19 and the neurological symptoms of LC, thus making it a relevant therapeutic target [125].

### 8.2. Spike-Based Adverse Vaccine Effects

Concerns regarding the adverse effects of vaccines (AEVs) have emerged from findings in cell-based assays and animal models utilizing the S protein and its S1/S2 subunits. Indeed, spike protein and the S1 subunit have been shown to mediate several cellular effects, including cell injury (e.g., oxidative stress, and paracrine senescence) and pro-inflammatory responses in several cell types in vitro (e.g., epithelial–endothelial cells and CNS cells) [80,81,126,127,128,129]. Furthermore, spike protein has also been shown to have deleterious effects in animal models, both at the CNS and lung levels [111,130]. Many of these findings are similar to those observed following the first BNT162b2 vaccination [131].

The AEVs may be associated with the pro-inflammatory effects of the mRNA encoding the S protein, subunits, and peptides, rather than with the potential pro-inflammatory effects of lipid nanoparticles (LNPs) [132,133] or the mRNA—in which uridine is replaced by pseudouridine—engineered to evade recognition by innate immune components [134]. However, AEVs may also be influenced by the vaccine platforms used, such as inactivated virus vaccines and S protein constructs, which include nanoparticles and viral vectors. Although rare, the AEVs induced by mRNA and vector vaccines may include serious clinical issues such as acute myocardial infarction, cerebral venous thrombosis, Guillain–Barré syndrome, myocarditis/pericarditis (primarily in younger individuals), as well as pulmonary embolism, stroke, thrombosis, herpes zoster reactivation, neurological complications, and autoimmune disorders [135,136,137,138,139]. It is important to note that certain adverse events following vaccination (AEVs), such as myocardial infarction and Guillain–Barré syndrome, are more commonly observed with increasing age, while others, like myocarditis, anaphylaxis, and appendicitis, are more prevalent among younger individuals [128,140]. Notably, myocarditis cases, although typically rare, have been observed at a higher frequency than expected after the second vaccine dose in both young boys and men in the US Army [141,142]. In fact, the presence of the S1 protein subunit was also detected in individuals who developed myocarditis, and coagulopathy, after vaccination [143]. Myalgic encephalomyelitis/chronic fatigue syndrome (ME/CSF) is among the most prevalent clinical conditions related to AEVs, and is also responsible for misdiagnosis, leading to delays and inappropriate treatment [144]. ME/CFS has also been documented after infection with other viruses, such as influenza and dengue viruses [145,146]. Therefore, to ensure an appropriate treatment, a correct diagnosis is critical, distinguishing the symptoms related to the vaccine from those arising from natural viral infections. Although there is overlap between the symptoms of acute COVID-19 and those that occur shortly after vaccination, certain distinguishing characteristics can be observed. For instance, the proportion of individuals experiencing AEVs is relatively low, reaching up to 5% [135,136,137]. Additionally, in cases of COVID-19, the most severe clinical outcomes leading to death typically occur within 1 to 3 weeks. In contrast, deaths associated with COVID-19 vaccination diminish exponentially over time after vaccination, usually resolving within about a week, though they may persist for up to a month [147,148,149]. Thus, a classification has been proposed based on the cause (infection/vaccination) and time of the manifestation of symptoms after vaccination [150]. Furthermore, SARS-CoV-2 mRNA sequence analysis from blood and tissue samples can help to identify the cause of disorders in affected patients, given that the spike mRNA COVID-19 vaccine sequences are only ~70% identical to the wild-type viral spike [151].

Although the molecular basis of AEVs remains unknown [135], the great overlap in symptoms caused by both severe COVID-19 and vaccination suggests that spike-mediated immune and vascular disorders may be a primary factor [152]. In this context, it is important to note that the mRNA-encoded S protein is modified to enhance expression efficiency and maintain stability for effective systemic distribution [134]. After the intramuscular injection of S protein encapsulated in LNPs, there is not only a local effect on the lymphatic system and spleen but also a significant release of the S protein and its fragments into the bloodstream [134]. Moreover, the S proteins can persist in circulation for extended periods, either within exosomes or through interactions with other circulating proteins, such as soluble ACE2 (sACE2) [153,154]. The persistence of the S protein and its byproducts may be a double-edged sword, accounting for both the robust and enduring systemic immune responses seen after vaccination [155,156] and potential adverse effects, such as the production of autoantibodies [137,157,158]. Additionally, it may disrupt the RAS by reducing both sACE2 and mACE2, which could trigger vasoconstriction, increased inflammation, and/or thrombosis [159] (Figure 1).

#### Vaccine Effect on Long COVID

Despite the growing body of evidence about vaccine adverse effects, prospective studies have shown that vaccination before or after SARS-CoV-2 infection reduces the risk of development of PASC-prolonged symptoms post-infection [160]. Up to two vaccination doses have been shown to reduce the risk of manifesting symptoms and mortality [20,161]. While the mechanisms behind vaccination-mediated long COVID protection or predisposition are not yet understood, the enhanced humoral (anti-S Abs) and cellular immune responses generated by vaccination may assist in the clearance of viral reservoirs and thus eliminate chronic inflammation caused by SARS-CoV-2 antigens. Despite the apparent protective effect of LC conferred by vaccines, it was recently demonstrated that high antibody titers induced by successive vaccinations may have opposite effects [162]. The latest findings on the positive and cumulative correlation between the number of vaccine doses and the prevalence of extended COVID-19 symptoms raise concerns, especially given the occurrence of successive waves of infection and the ongoing global vaccination programs. Therefore, it may be prudent to administer booster doses only in cases where the benefit–risk profile is clearly established, such as for the elderly and patients with comorbidities. In this context, prioritizing a deeper understanding of the molecular basis underlying adverse events is essential, along with conducting long-term clinical studies comparing vaccinated individuals with controls to identify potential biomarkers.

## 9. Conclusions Remarks and Future Directions

The rapid development of vaccines helped to mitigate the devastating effects of the COVID-19 pandemic. Nevertheless, the emergence of LC and the spread of VCOs showing reduced susceptibility to vaccination and immunity from prior infections represent a new challenge. In this scenario, the spike protein emerges as a relevant factor in long COVID’s pathophysiological mechanisms, such as the persistence of viral component reservoirs, endothelial dysfunction and thromboinflammation, immune dysregulation/autoimmunity, and complement activation. In this context, it is essential to conduct further studies to assess the impact of the S protein on each pathophysiological mechanism related to LC, as well as the specific molecular mechanisms by which the S protein may influence clinical outcomes.

## Abbreviations

PASC	Post-acute sequelae of COVID-19
ICU	Intensive care unit
TNF-α	Tumor necrosis factors alpha
IL-1β	Interleukin 1 beta
IL-6	Interleukin 6
IL-18	Interleukin 18
NF-kB	Nuclear factor kappa B
IL-6R	Interleukin 6 receptors
ADAM17	A disintegrin and metalloprotease 17
ICAM-1	Intercellular adhesion molecules 1
VCAM-1	Vascular cell adhesion molecules 1
MCP-1	Monocyte chemoattractant protein 1
VAP-1	Vascular adhesion protein 1
AT1R	Angiotensin 1 receptor
MDM2	Murine doble minute 2
IFN	Interferon
TLR2/4	Toll-like receptor 2/4
EBNA1	Epstein–Barr nuclear antigen 1
