# Correction: de Melo et al. SARS-CoV-2 Spike Protein and Long COVID—Part 1: Impact of Spike Protein in Pathophysiological Mechanisms of Long COVID Syndrome. *Viruses* 2025, *17*, 617

**DOI:** 10.3390/v18010002

**Published:** 2025-12-19

**Authors:** Bruno Pereira de Melo, Jhéssica Adriane Mello da Silva, Mariana Alves Rodrigues, Julys da Fonseca Palmeira, Felipe Saldanha-Araujo, Gustavo Adolfo Argañaraz, Enrique Roberto Argañaraz

**Affiliations:** 1 Laboratory of Molecular Neurovirology, Department of Pharmacy, Faculty of Health Science, University of Brasília, Brasilia 70910-900, DF, Brazil; 2 Laboratory of Hematology and Stem Cells (LHCT), Faculty of Health Sciences, University of Brasília, Brasilia 70910-900, DF, Brazil

**Error in Funding**

In the original publication [1], the funding from the Federal District Research Support Foundation (FAPDF), DPI/BCE 01/2025, and the Bench Amendment, No. 7108001, was not included. The correct funding information should be as follows:

“This study received financial support from the Federal District Research Support Foundation (FAPDF), DPI/BCE 01/2025, and through Bench Amendment No. 7108001.” 

The authors state that the scientific conclusions are unaffected. This correction was approved by the Academic Editor. The original publication has also been updated.
